# Dexmedetomidine use during orthotopic liver transplantation surgery on early allograft dysfunction: a randomized controlled trial

**DOI:** 10.1097/JS9.0000000000001669

**Published:** 2024-05-20

**Authors:** Liqun Yang, Ling Zhu, Bo Qi, Yin Zhang, Chenlu Ni, Yijue Zhang, Xiao Shi, Qiang Xia, Joe Masters, Daqing Ma, Weifeng Yu

**Affiliations:** a Department of Anesthesiology, Renji Hospital, Shanghai Jiao Tong University School of Medicine, Shanghai; Key Laboratory of Anesthesiology (Shanghai Jiao Tong University), Ministry of Education; b Department of Hepatology, Renji Hospital, Shanghai Jiao Tong University School of Medicine, Shanghai, People’s Republic of China; c Division of Anesthetics, Pain Medicine & Intensive Care, Department of Surgery and Cancer, Faculty of Medicine, Imperial College London, Chelsea and Westminster Hospital, London, United Kingdom; d Perioperative and Systems Medicine Laboratory, Children’s Hospital, Zhejiang University School of Medicine, National Clinical Research Center for Child Health, Hangzhou, China

**Keywords:** dexmedetomidine, early allograft dysfunction, orthotopic liver transplantation, primary graft dysfunction

## Abstract

**Background:**

Previous studies have shown a protective effect of dexmedetomidine use in kidney transplantation. In contrast, it is not known whether intraoperative administration of dexmedetomidine can reduce early allograft dysfunction (EAD) incidence following liver transplantation.

**Objective:**

To investigate the effect of dexmedetomidine use during surgery on EAD following orthotopic liver transplantation (OLT).

**Study Design:**

This is a single-center, double-blinded, placebo-controlled randomized clinical trial. Three hundred thirty adult patients undergoing OLT were enrolled from 14th January 2019 to 22nd May 2022. Patients received dexmedetomidine or normal saline during surgery. One year follow-ups were recorded.

**Methods:**

Patients were randomized to two groups receiving either dexmedetomidine or normal saline intraoperatively. For patients in the dexmedetomidine group, a loading dose (1 μg/kg over 10 min) of dexmedetomidine was given after induction of anesthesia followed by a continuous infusion (0.5 μg/kg /h) until the end of surgery. For patients in the normal saline group, an equal volume loading dose of 0.9% saline was given after the induction of anesthesia followed by an equal volume continuous infusion until the end of surgery. The primary outcome was EAD. Secondary outcomes included primary graft nonfunction, acute kidney injury, and acute lung injury/acute respiratory distress syndrome.

**Results:**

Of 330 patients included in the intention-to-treat analysis, 165 were in the dexmedetomidine group [mean (SD) age, 49 (10) years; 117 (70.9%) men], and 165 were in the normal saline group [mean SD age, 49 (9) years; 118 (74%) men]. 39 (24.4%) patients in the dexmedetomidine group and 31 (19.4%) in normal saline group developed EAD and the difference was statistically insignificant (*P*=0.28). Secondary outcomes including primary graft nonfunction and acute kidney injury was similar between the two groups.

**Conclusion:**

Intraoperative administration of dexmedetomidine did not reduce EAD rate after OLT.

## Introduction

Highlights

- Early allograft dysfunction is a common and severe complication following liver transplantation.
- Although laboratory studies have demonstrated the hepatoprotective quality of dexmedetomidine, clinical evidence remains scarce.
- Intraoperative dexmedetomidine did not reduce the occurrence of early allograft dysfunction in adult liver transplant recipients.

Orthotopic liver transplantation (OLT) is a potentially life-saving treatment for patients with fulminant liver failure and end-stage liver diseases^1^. Early allograft dysfunction (EAD), a term used to define initial poor graft function within 1 week after OLT, poses serious challenges to the success of transplant surgery and patient survival^2,3^. It has been estimated that EAD occurs in over 20–44% patients after surgery^4–6^. Previous work suggested that using remote ischemia preconditioning^7–9^ and hypothermic oxygenated machine perfusion^10–12^ to preserve grafts were beneficial. However, those strategies are either inconvenient to implement or require additional equipment. There is still a need for more convenient and feasible strategies to improve allograft outcomes.

Dexmedetomidine, a highly potent α_2_-agonist, is widely used as a sedative among major surgeries^13,14^. The protective effect of dexmedetomidine has been reported in ischemia-reperfusion injury in organs including heart, liver, kidney, intestine, and brain^15–18^. The effect may stem from its anti-inflammatory properties as demonstrated by decreased IL-1β, IL-6, and TNF-α levels^19^ in animal models^20,21^. Clinical evidence on the topic is scarce, especially in liver transplantation surgery. Several clinical studies have reached the conclusion that dexmedetomidine exerts a protective effect after hepatectomy^13,21,22^. However, this protection was mainly evidenced by improved biomarkers of liver function within the first postoperative day^13^.

In this single-center, double-blind, placebo-controlled randomized clinical trial, we investigated the effect of dexmedetomidine on EAD following orthotopic liver transplantion. We hypothesized that application of dexmedetomidine throughout the surgical procedure may help reduce the incidence of early graft dysfunction.

## Methods

### Trial design and ethics

**Figure 1:**
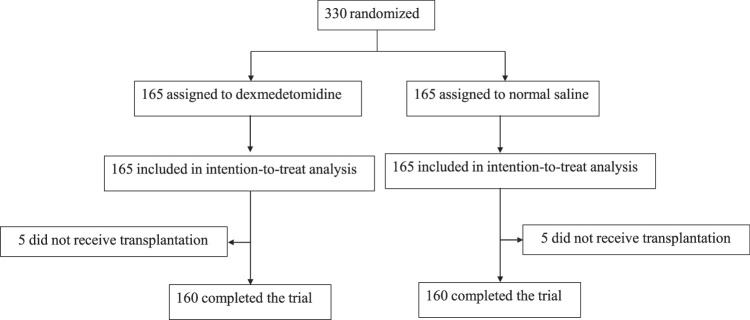
Trial flowchart.

Consolidated Standards of Reporting Trials (CONSORT, Supplemental Digital Content 1, http://links.lww.com/JS9/C628) reporting guideline was followed. After written informed consent obtained, 330 patients were enrolled from January 2019 and May 2022 (Fig. 1). Study protocol was previously published^23^ and is provided in Supplement 1 (Supplemental Digital Content 2, http://links.lww.com/JS9/C629). Written informed consent was obtained from all patients.

## Participants

### Inclusion and exclusion criteria

Patients aged between 18 and 65 undergoing allogenic liver transplant surgery under general anesthesia, who meet the UCSF criteria (single tumor diameter ≤6.5 cm; multiple tumors ≤3, maximum diameter ≤4.5 cm; cumulative diameter ≤8 cm; without large vessel infiltration and extrahepatic metastasis) were eligible for trial inclusion^24^.

Patients with severe renal dysfunction (undergoing renal replacement therapy before surgery), severe pulmonary dysfunction (severe pre-existing chronic lung disease), severe circulatory instability [severe coronary artery disease, unstable angina, left ventricular ejection fraction <30%, sick sinus syndrome, severe sinus bradycardia (<50 bpm), second-degree or greater atrioventricular block], known allergy or intolerance to trial medication, participation in other clinical trials within 30 days prior to randomization, retransplantation, and multiple organ transplantation were excluded.

### Outcomes

The primary outcome was the incidence of EAD following OLT surgery, which is defined according to Olthoff’s criteria published in 2010: (1) bilirubin ≥10 mg/dl on day 7; or (2) INR >1.6 on day 7; or (3) AST/ALT >2000 IU/l within first 7 days^25^.

Secondary outcomes included incidence of primary nonfunction (PNF), incidence of postoperative acute kidney injury (AKI), and acute respiratory distress syndrome (ARDS) PNF was defined as graft loss, retransplantation, or participant’s death due to graft nonfunction in first 30 days (excluding nonfunction secondary to hepatic artery thrombosis, biliary complications, or recurrent hepatic disease). AKI was defined by Kidney Disease: Improving Global Outcomes (KDIGO) Criteria published in 2012 and ARDS was defined according to Berlin modification of the American European Consensus Committee (AECC) definitions published in 2012.

### Anesthesia and intervention

All participants received standard anesthesia care according to our hospital’s protocol. Anesthesia was induced using propofol, sufentanil, and rocuronium/cisatracurium for neuromuscular blockage. Remifentanil, propofol, and sevoflurane/desflurane were used for anesthesia maintenance. Mechanical ventilation was performed following routine protocol, in which tidal volume was kept between 6 and 8 ml/kg, positive end expiratory pressure (PEEP) at between 5 and 8 cmH_2_0, plateau pressure of less than 30 cmH_2_0, ventilation frequency between 10 and 16 per minute, and 1:1 air-oxygen mixture. Intraoperative fluid therapy was managed according to the anesthesiologist’s routine practice. Hemoglobin levels were maintained within the range of 7–10 g/dl. Transfusion and vasopressors were given when the anesthesiologist decided. Intravenous steroids were administered before reperfusion of the new liver as per normal protocol.

For the dexmedetomidine group, patients received an initial loading dose of dexmedetomidine of 1 μg/kg over 10 min after the induction of anesthesia followed by a continuous infusion of 0.5 μg/kg/h until the end of surgery. In the normal saline group, participants received a same volume of saline instead of dexmedetomidine.

### Allograft and surgery

Organ donation or transplantation in the study was strictly implemented under the regulation of Organ Transplant Committee and the Declaration of Helsinki. Ethical approval was obtained from the Committee of Ethics at the hospital. Cadaveric donors involved in the study were brain-dead donors. Donors were preserved in conventional static cold preservation and transported to our medical center. All the surgical procedures were performed by specialists with experience in the OLT technique. Classic OLT was the only surgical technique and postoperative care were given following our routine protocol.

### Sample size calculation

The sample size was calculated based on the incidence of EAD was reported to be up to 35%^2,6^. If dexmedetomidine administration reduces the incidence by 15%, 136 participants in each group are required to have an 80% power at the two-sided 0.05 significance level. With an anticipated 20% dropout rate, a total of 165 participants for each group are required. Accordingly, the effect size is 0.31, which is also in line with the general recommendations to detect a medium effect size.

### Randomization and blinding

An independent statistician generated a random sequence of 0 and 1 in a 1:1 fashion correspondent to numbers from 1 to 330. Participants who consented to enter the trial were sequentially assigned a random number allocating them to either the dexmedetomidine or normal saline group. The treatment drugs were labeled with unique codes that were known only to an independent pharmacist, ensuring that caregivers remained blinded. Besides participants and researchers, outcome assessors responsible for evaluate outcomes and collect data were also blinded to the treatment assignments.

### Statistical analysis

All analyses were conducted based on the intention-to-treat principle. Numeric variables including postoperative laboratory results are presented as mean (SD) or median (minimum, maximum; or interquartile range) where appropriate. Categorical variables including the incidence of EAD, PNF, AKI, and ARDS after OLT are presented as number of cases (percentage). Difference of numeric variables between groups are analyzed using independent sample *t*-test, and that of categorical variables are analyzed using *χ* ^2^ test. Results are reported as hazard ratios and 95% CI. Two-tailed *P*-values of less than 0.05 are considered to be of statistical significance.

A *post-hoc* analysis of treatment effect heterogeneity included the following subgroups: (1) age >50 versus ≤50 years old; (2) sex; (3) Child-Pugh Score; (4) MELD Score; (5) Portal occlusion time ; (6) graft cold ischemia time; (7) graft condition and RBC transfusion. For each subgroup, covariate-by-treatment interaction was tested using log-binomial regression.

## Results

One hundred sixty-five per group were included in intention-to-treat analysis (Fig. 1). Five patients from the dexmedetomidine group and five from the normal saline group did not receive OLT surgery due to various reasons including those grafts were not suitable for engraftment, leaving 160 per group completed the trial.

The median age was 49 years in both the groups, and most were men (Table 1). About 70% were evaluated with an ASA score of 3. Mean Child-Pugh score was six in dexmedetomidine group and seven in normal saline group. Mean MELD score was 13 in both groups. 65% in the dexmedetomidine group and 71.3% in normal saline group were diagnosed with chronic hepatitis, and 34.4% and 38.8%, respectively, were with hepatic cancer. The most common comorbidity was diabetes in both groups. All grafts were retrieved from deceased donors. Average cold ischemia time of allografts were 8.2 h in both groups. Most donors died from head trauma or stroke. Over 20% transplants were burdened by steatosis.

**Table 1:** Baseline patient characteristics.

	dexmedetomidine (*N*=165 )	normal saline (*N*=165)
Recipient characteristics		
Age, mean (SD), year	49 (10)	49 (9)
Sex, *n* (%)		
Men	117 (70.9)	122 (73.9)
BMI, mean (SD)	23.4 (3.6)	23.5 (4.0)
ASA classification level, *n* (%)*		
II	32 (20.0)	36 (22.5)
III	111 (69.4)	112 (70.0)
IV	8 (5.0)	4 (2.5)
Child-Pugh score, median (IQR)	6 (5–8)	7 (5–8)
MELD score, median (IQR)	13 (10–18)	13 (9–18)
Primary diagnosis, *n* (%)		
Chronic hepatitis	104 (65.0)	114 (71.3)
Hepatic cancer	55 (34.4)	62 (38.8)
Alcoholic cirrhosis	10 (6.3)	7 (4.4)
Cholestatic disease	3 (1.9)	5 (3.1)
Metabolic disease	6 (3.8)	4 (2.5)
Other	23 (14.4)	35 (21.9)
Comorbidity, *n* (%)		
Anaphylactic history	11 (6.9)	12 (7.5)
Hypertension	7 (4.4)	15 (9.4)
Diabetes	15 (9.4)	16 (10.0)
Kidney disorder	2 (1.3)	3 (1.9)
other	6 (3.8)	5 (3.1)
Preoperation laboratory data		
Hemoglobin,g/l,mean (SD)	109 (32.4)	111 (32.5)
ALT,U/l,median (IQR)	31 (22–51)	33 (23–54)
AST,U/l,median (IQR)	41 (29–86)	43 (31–75)
Prothrombin time,s,median (IQR)	15 (13–17)	15 (13–17)
Platelets,10^9^/L,U/L,median (IQR)	77 (46–123)	72 (44–121)
Total bilirubin,μmol/L, median (IQR)	38 (21–73)	33 (18–93)
White blood cell level,median (IQR)	4 (3–6)	4 (3–6)
Neutrophil percentage,median (IQR)	66 (56–75)	65 (54–72)
Albumin level,mean (SD),g/l	37 (8.7)	35 (8.6)
Creatinine level,median (IQR),μmol/l	66 (54–79)	61 (48–77)
Blood glucose level,median (IQR),mmol/l	5.2 (4.5–6.4)	5.1 (4.2–6.4)
Donor characteristics		
Cold ischemia time,median (IQR),h	8.2 (6.8–10.7)	8.2 (5.9–10.3)
DCD, *n* (%)	165 (1.0)	165 (1.0)
Reason for death, *n* (%)		
Head trauma	51 (31.9)	39 (24.4)
Stroke	66 (41.3)	66 (41.3)
Cardiac arrest	1 (0.6)	1 (0.6)
Car accident	5 (3.1)	6 (3.8)
Other	8 (5.0)	10 (6.3)
Donor liver condition		
HBV	9 (5.6)	3 (1.9)
Liver steatosis	46 (28.8)	33 (20.6)
Cholestatic disease	1 (0.6)	2 (1.3)
Other	14 (8.8)	25 (15.6)

* Data missing in some patients.

There were no significant between-group differences in anesthesia time, surgery time, portal occlusion time, vena cava occlusion time (Table 2). Fluid and blood product transfusion were comparable between the two groups.

**Table 2:** Intraoperative characteristics.

	Dexmedetomidine (*N*=160)	Normal saline (*N*=160)	Mean difference (95% CI)	*P*
Intraoperativie variable				
Anesthesia time,median (IQR), min	399 (353–458)	409 (364–455)	(−23.60–10.29)	0.41
Surgery time,median (IQR),min	337 (297–397)	349 (304–392)		0.70
Portal occlusion time,median (IQR),min	40 (36–48)	42 (35–47)		0.58
Vena cava occlusion time,median (IQR),min	38 (34–46)	38 (33–43)		0.60
Crystalloid solution,median (IQR),l	2.0 (1.5–2.5)	2.0 (1.5–2.5)	(−0.21–0.12)	0.19
Colloid solution,median (IQR),l	1.3 (1.0–1.8)	1.3 (1.0–1.8)	(−0.14–0.10)	0.46
Blood loss during surgery,median (IQR),l	0.6 (0.4–0.8)	0.6 (0.4–1.0)		0.80
Ascites amount, median (IQR), l	1.0 (0.5–2.3)	1.0 (0.2–2.0)		0.43

In the ITT analysis, EAD occurred in 39 (24.4%) patients assigned to dexmedetomidine group, and 31 (19.4%) assigned to normal saline group and the difference was not statistically significant (*P*=0.28). Four patients in dexmedetomidine group and four in normal saline group developed PNF. Forty-eight (30.0%) patients in dexmedetomidine group, and 60 (37.5%) in normal saline group had AKI (*P*=0.16). ALI/ARDS events were statistically comparable between the two groups [29 (18.1%) vs. 18 (11.3%), *P*=0.08]. Other outcomes including postoperative medication, cardiac events, ICU admission and stay, hospital stay were also statistically comparable between the two groups. One year follow-up visits found no differences in retransplantation and death (Table 3). Intraoperative dexmedetomidine and normal saline also resulted in comparable patients’ surviaval probability with 1-year after surgery (*P*=0.45) (Fig. 3).

**Table 3:** Primary and secondary outcomes.

	dexmedetomidine (*N*=160)	Normal saline (*N*=160)	Relative risk (95% CI)	*P*
Primary outcomes				
EAD, *n* (%)	39 (24.4)	31 (19.4)	0.80 (0.52–1.21)	0.28
Secondary outcomes				
PNF, *n* (%)	4 (2.5)	4 (2.5)	1.00 (0.25–3.39)	0.31
AKI, *n* (%)	48 (30.0)	60 (37.5)	1.25 (0.92–1.70)	0.16
ALI/ARDS, *n* (%)	29 (18.1)	18 (11.3)	0.62 (0.36–1.07)	0.08
Postoperative medication, *n* (%)				
Sedatives	31 (19.4)	29 (18.1)	0.94 (0.59–1.48)	0.78
Analgesics	92 (57.5)	90 (56.3)	0.98 (0.81–1.18)	0.82
Anticholinergic agents	7 (4.4)	3 (1.9)	0.43 (0.11–1.63)	0.20
Steroids	155 (96.9)	150 (93.8)	0.97 (0.92–1.02)	0.19
Postoperative complications, *n* (%)				
Hypertension	1 (0.6)	1 (0.6)	1.00 (0.06–15.85)	1.00
Hypotension	0 (0)	2 (1.25)	0.99 (0.97–1.01)	0.50
Arrythmia	0 (0)	4 (2.5)	0.98 (0.95–1.00)	0.13
Acute myocardial infarction	0 (0)	1 (0.6)	0.99 (0.98–1.01)	1.00
Atelectasis	23 (14.4)	16 (10.0)	0.70 (0.38–1.27)	0.23
Pneumonia	87 (54.4)	83 (51.9)	0.95 (0.78–1.17)	0.65
Pneumothorax	0 (0)	1 (0.6)	0.99(0.98–1.01)	1.00
Hydrothorax	143 (89.4)	128 (80.0)	0.90 (0.82–0.98)	0.02
Anastomotic fistula	1 (0.6)	1 (0.6)	1.00 (0.06–15.85)	1.00
Abdominal abscess	5 (3.1)	2 (1.3)	0.40 (0.08–2.03)	0.45
Active bleeding	3 (1.9)	1 (0.6)	0.33 (0.04–3.17)	0.62
Thrombosis	1 (0.6)	2 (1.3)	2.00 (0.18–21.84)	1.00
ICU admission, *n* (%)	154 (96.2)	153 (95.6)	0.99 (0.95–1.04)	0.78
ICU stay, median (IQR), d	2 (1.0)	3 (1.0)	1.50 (0.25–8.86)	1.00
Reintubation, n.(%)	12 (7.5)	7 (4.4)	0.58 (0.24–1.44)	0.24
Reoperation, *n* (%)	15 (9.4)	10 (6.3)	0.67 (0.31–1.44)	0.30

**Figure 2:**
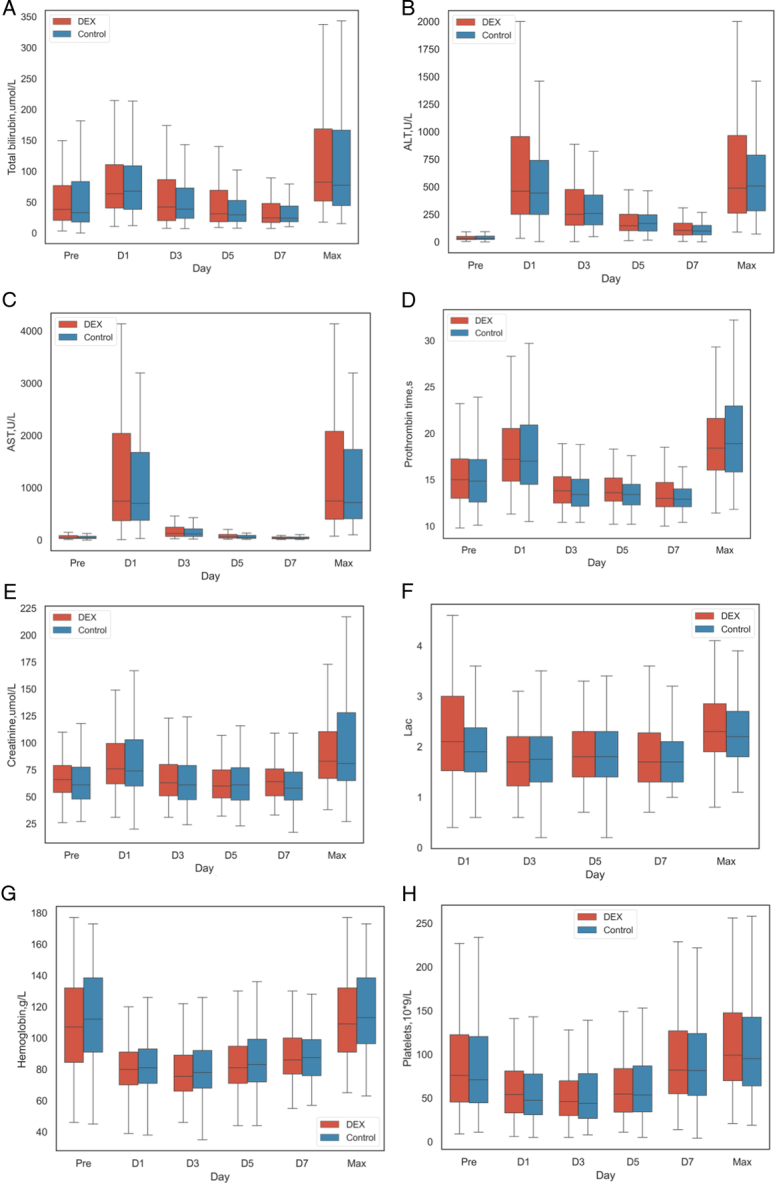
Serial changes in perioperiatvie laboratory data in recipients. A-H There were no significant differences in total bilirubin, ALT, AST, creatinine, lactic acid, hemoglubin, and platelets level between the two groups (Fig. 2).

**Figure 3:**
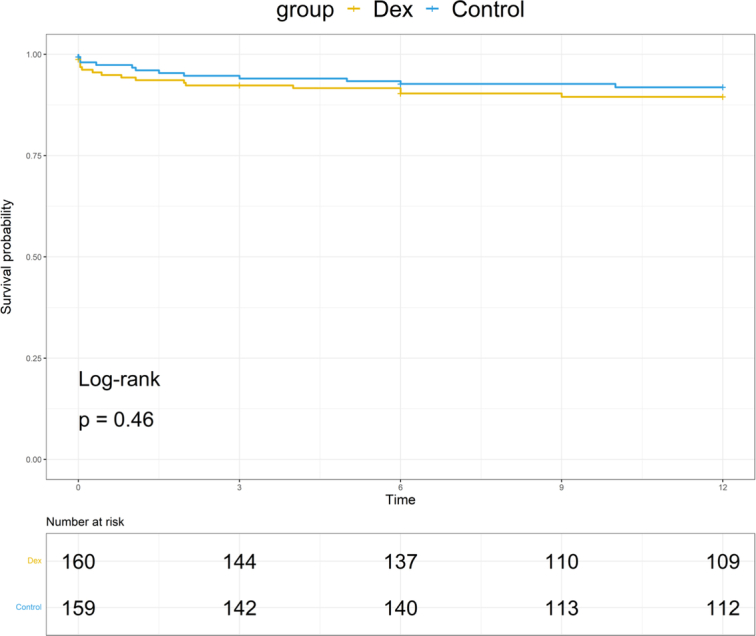
Recipients survival. Probability within 1 year postoperatively.

**Figure 4:**
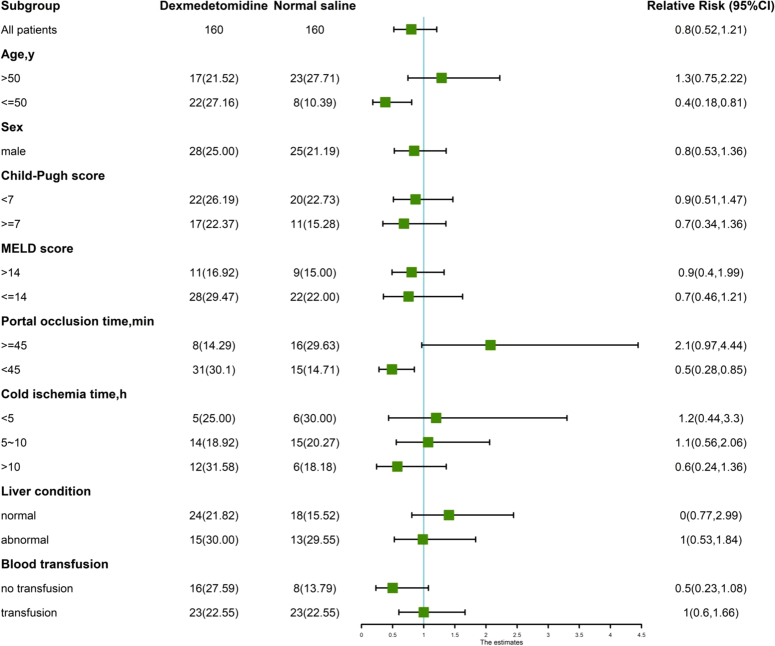
Subgroups analysis of treatment effect heterogeneity.

Subgroup analyses demonstrated reduced EAD within the dexmedetomidine group among patients aged ≤50 years, or whose portal occlusion time less than 45 min, relative risk were 0.38 (95% CI: 0.18–0.81) and 0.49 (0.28–0.85), respectively. However, there was no evidence of heterogeneity of effects across subgroup defined by sex, MELD score, allograft condition, cold ischemia time, and transfusion (Fig. 4).

## Discussion

In this randomized trial, patients who received intraoperative dexmedetomidine did not reduce EAD after liver transplantation compared with normal saline. Furthermore, there were no statistically significant difference in postoperative PNF, AKI, or ALI/ARDS between dexmedetomidine and normal saline. This study does not provide evidence that dexmedetomidine exerts any benefits when compared to placebo in liver transplantation. To our knowledge, this is the largest trial by far investigating the effect of dexmedetomidine on allograft outcomes after adult orthotopic liver transplantion.

EAD is a major complication after surgery, occurs in one of every five patients^25^. Many factors contribute to the occurrence of EAD during the perioperative period: including extended criteria donors, cold preservation, and unavoidable ischemia injury during surgery^26^. Development of EAD negatively impacts outcome, eventually leading to PNF, urgent retransplantation or even death^27^. In the present trial, incidence of EAD is around 20%, in line with previously reported. Reported efforts to decrease EAD include minimizing ischemia-reperfusion injury, machine perfusion, preventative medications. Substances like rifaximin^28^ and omega-3 fatty acids^29^ have been investigated to protect liver function, but none has yielded positive outcomes in clinical studies thus far. Thereafter, administration of organ protective drug perioperatively might be a promising and feasible approach to reduce the incidence of EAD.

Since approved by the Food and Drug Administration (FDA) for use during general anesthesia in 2009^30^, dexmedetomidine has been widely used during major surgeries because of its anxiolytic, analgesic, and sedative properties. Dexmedetomidine exhibits protective effect in multiple organs, including liver, kidney, lung, heart, brain, and intestine^30^. Moreover, its protective effect has been reported in kidney transplantation as well. In a trial of 114 patients, dexmedetomidine reduced the incidence of delayed graft function after kidney transplant, as well as decreased need for dialysis^31^. Nonetheless, in the context of liver transplantation, the risk-benefit profile of dexmedetomidine had not been fully assessed. In preclinical studies, dexmedetomidine was reported to have anti-inflammative, antioxidative and antiapoptotic properties^30^. In a rat orthotopic autologous liver transplantation model, pretreatment with dexmedetomidine increased levels of antioxidants, attenuated renal injury^32^ and mitigated liver injury via suppression of TLR4/NF-κB pathway^21^. Nevertheless, further clinical substantiation of its impact on liver transplant surgery is limited.

Several previous studies highlighted the potential benefits of dexmedetomidine in liver transplantation. But these studies were with small sample size and divergent primary outcomes. For instance, a study involving 49 liver recipients found that dexmedetomidine group was associated with shorter ICU stay^33^. Similarly, in another trial of 40 adult living donor liver transplantation, dexmedetomidine exerted protective effects against hepatic IRI, as evidenced by reduced ICAM-1 level, and minimal histopathological changes^34^. Our study has enrolled 330 participants and adopted EAD as primary study outcome, which identifies OLT allografts with initial poor function and also portends poor allograft and patient survival. Regrettably, our study did not reveal any beneficial effects of dexmedetomidine on graft outcomes.

This study has several limitations. Firstly, this is a single-center study, which inherently limits its applicability to a broader context. Secondly, the dosing of the study drug was determined according to our hospital’s practice routine, given the absence of prior relevant trials. Worth-mentioning is the fact that our hospital is one of world’s largest liver transplantation center, and our team is the primary anesthesia team for these procedures. The dosage employed in this study is considerably lower than that reported in animal studies when adjusted based on body weight^20^. Thirdly, administration of dexmedetomidine is restricted only to intraoperative period rather than extending to postopetrative phase. Consequently, its influence on liver grafts may have been constrained. Fourthly, plasma concentration of dexmedetomidine is not monitored. Dexmedetomidine undergoes metabolism primarily in the liver, therefore hepatic failure can notably influence plasma levels of the drug. Additionally, dexmedetomidine binds strongly to plasma proteins (94%) in the circulation. Patients with reduced liver function are likely to exhibit lower drug binding affinity and slower clearance rate^35^. Patients undergoing OLT surgery are likely to have impaired liver function, which could lead to prolonged drug elimination half-life and decreased clearance rate. Hence, its ‘toxic’ over protective effects may also be possible *per se*.

## Conclusion

In this randomized clinical trial, intraoperative dexmedetomidine did not reduce EAD rate after adult liver transplantation. The benefits of the use of dexmedetomidine in OLT are subjected for further study.

## Ethical approval

This randomized controlled trial was approved by the ethics committee of Renji Hospital, Shanghai Jiao Tong University. Reference number: 2018-022.

## Consent

Written informed consent was obtained from the patient for publication of this case report and accompanying images. A copy of the written consent is available for review by the Editor-in-Chief of this journal on request.

## Sources of funding

This study was funded by Pudong New Area Health Commission (No. PW2022D-01), the Science and Technology Commission of Shanghai Municipality (No. 20410760500), the National Natural Science Foundation of China (No. 82371517, 81771185, U23A20508, 82300725), Shanghai Municipal Key Clinical Specialty (No. shslczdzk03601), Innovation Program of Shanghai Municipal Education Commission (No. 2019-01-07-00-01-E00074), Shanghai Engineering Research center of Perioperative Organ Support and Function Preservation (No. 20DZ2254200) and the Scientific Research Project of Shanghai Municipal Commission of Health (No. 202040013). The funders did not participate in study design, data collection, data analysis, data interpretation, writing or approval of the report, nor the decision to submit for publication. The corresponding authors have full access to all trial data and take final responsibility for the decision to submit for publication.

## Author contribution

L.Y., D.M., and W.Y.: conceptualization; Y.Z.: formal analysis; L.Y., B.Q., and W.Y.: funding acquisition; L.Z., B.Q., Y.Z., C.N., and X.S.: investigation; D.M. and J.M.: methodology; Q.X.: resources; L.Z.: writing – original draft; L.Y. and D.M.: writing – review and editing.

## Conflicts of interest disclosure

All authors declare no competing interests.

## Research registration unique identifying number (UIN)

ClinicalTrials.gov NCT03770130. Registered on 10 December 2018. https://clinicaltrials.gov/ct2/show/NCT03770130.

## Guarantor

Liqun Yang and Weifeng Yu.

## Data availability statement

Original data available upon request to the corresponding authors. Access to the data that supports the finding of this study is provided upon request, and researchers can obtain the dataset by contacting Dr Daqing Ma at d.ma@imperial.ac.uk, or Dr Weifeng Yu at ywf808@yeah.net. Data will only be provided for research purpose. In order to protect patient privacy, only deidentified data will be provided.

## Provenance and peer review

This paper is not invited.

## Supplementary Material
