# Supplementary material for: Dexmedetomidine use during orthotopic liver transplantation surgery on early allograft dysfunction: a randomized controlled trial
Source: Int J Surg. 2024 May 20;110(9):5518–26. doi: 10.1097/JS9.0000000000001669 (PMC11392095; doi:10.1097/JS9.0000000000001669)
Supplement: Supplementary file 2 [file js9-110-5518-s002.docx]

Study Protocol

Dexmedetomidine use during orthotopic liver transplantation surgery on early allograft dysfunction: A randomized controlled trial

**Purpose**

Earlier investigations have demonstrated the advantageous impact of employing dexmedetomidine during kidney transplantation, as it exerts a protective influence. However, it remains unclear whether the intraoperative administration of dexmedetomidine can diminish the occurrence of early allograft dysfunction after liver transplantation.

The purpose of this single-center, double-blinded, placebo-controlled randomized clinical trial is to evaluate the effect of intraoperative dexmedetomidine on early graft dysfunction after orthotopic liver transplant.

**Study summary**

**Title**

Dexmedetomidine use during dexmedetomidine liver transplantation surgery on early allograft dysfunction: A randomized controlled trial

**Objective**

To investigate the effect of dexmedetomidine use during surgery on early allograft dysfunction following liver transplantation.

**Inclusion criteria**

Patients aged between 18 to 65 undergoing allogenic liver transplant surgery under general anesthesia, who meet the UCSF criteria (single tumor diameter ≤ 6.5 cm; multiple tumors ≤ 3, maximum diameter ≤ 4.5 cm; cumulative diameter ≤ 8 cm; without large vessel infiltration and extrahepatic metastasis) were eligible for trial inclusion.

**Exclusion criteria**

Patients with severe renal dysfunction (undergoing renal replacement therapy before surgery), severe pulmonary dysfunction (severe pre-existing chronic lung disease), severe circulatory instability (severe coronary artery disease, unstable angina, left ventricular ejection fraction < 30%, sick sinus syndrome, severe sinus bradycardia [< 50 bpm], second-degree or greater atrioventricular block), known allergy or intolerance to trial medication, participation in other clinical trials within 30 days prior to randomization, re-transplantation and multiple organ transplantation were excluded.

**Drop out criteria**

Consents may be withdrawn by the participants or their legal representative. Loss to follow-up is also considered drop out.

**Outcomes**

The primary outcome was the incidence of EAD following surgery.

Secondary outcomes included incidence of primary nonfunction (PNF), incidence of postoperative acute kidney injury (AKI) and acute respiratory distress syndrome (ARDS) during postoperative day 1-7, incidence of graft failure and re-transplantation ratio during 1-year follow-up period, 1-year all-cause mortality rate. PNF was defined as graft loss, re-transplantation, or participant’s death due to graft non-function in first 30 days (excluding non-function secondary to hepatic artery thrombosis, biliary complications, or recurrent hepatic disease). AKI was defined by Kidney Disease: Improving Global Outcomes (KDIGO) criteria published in 2012 and ARDS was defined according to Berlin modification of the American European Consensus Committee (AECC) definitions published in 2012.

**Study design**

Single-center, double-blinded, placebo-controlled randomized clinical trial

Patients were randomized to two groups receiving either dexmedetomidine or placebo intraoperatively. For patients in the dexmedetomidine group, a loading dose (1 μg/kg over 10 min) of dexmedetomidine was given after induction of anesthesia followed by a continuous infusion (0.5 μg/kg /h) until the end of surgery. For patients in the placebo group, an equal volume loading dose of 0.9% saline was given after the induction of anesthesia followed by an equal volume continuous infusion until the end of surgery. Other clinical intervention followed routine hospital protocol.

Study outcomes were measured on day 1,3,7,14,30 and before discharge. Follow-up visits were paid via phone at 3, 6, and 12 months.

**Study institution**

Ren Ji Hospital, Shanghai Jiao Tong University School of Medicine, Shanghai 200127, China

**Significance**

Hepatic failure is a condition that carries significant morbidity and mortality rates. Decompensated cirrhosis with the development of ascites has a 2-year survival rate of only around 50% ^1^. In 2015, cirrhosis affected an estimated 2.8 million individuals worldwide and resulted in 1.8 million deaths ^2,3^. Hepatic failure also has wide-ranging effects on various organ systems, including the cardiovascular, pulmonary, renal, and neurological systems. Orthotopic Liver transplantation (OLT) is considered the most effective treatment for individuals with end-stage liver disease and offers a significant survival benefit. Many centers report a 1-year survival rate of 85-95% and a 5-year survival rate of 70-80% ^4,5^.

Early allograft dysfunction (EAD) is a term used to define initial poor graft function after transplantation^6^. The consequences of early allograft dysfunction can be significant, leading to prolonged hospital stays, increased risk of infections, higher rates of rejection, and even graft loss^7,8^. It has been estimated that EAD occurs in over 20% to 44% patients after surgery^9-11^.

**Background**

Due to a widespread shortage of donor organs, there has been an increase in the acceptance of organs of marginal quality that are particularly prone to perioperative injury and are at increased risk of early postoperative dysfunction and failure ^12,13^. There is therefore significant interest in the development of new strategies to prevent liver graft dysfunction and early graft loss.

Initial dysfunction of the liver graft in the immediate postoperative period is indicative of hepatocellular damage and synthetic impairment and is strongly predictive of poor graft and patient survival ^14-16^. EAD and Primary nonfunction (PNF) are two forms of graft dysfunction after liver transplantation. EAD serves as an important surrogate end point in OLT clinical trials. The incidence of EAD is approximately 25% in most large studies, although reports range from 9.3 to 43.7% ^4,10,15,17^. PNF occurs in up to 7% of grafts and requires urgent re-transplantation ^4^.

Graft dysfunction is multifactorial; among other factors, donor and recipient characteristics play a role. Currently, no treatment to prevent EAD or PNF is available. Choice of anesthetic drugs for liver transplantation anesthesia for OLT has evolved significantly since the first transplants in the 1960s, with increasing recognition that the anesthetist plays a key role in influencing outcomes (such as graft function and survival) by optimizing parameters such as the patient’s hemodynamic status, coagulation profile, and volume status. In addition to this, it has been postulated that the type of anesthetic drugs administered in the perioperative period may also affect outcomes ^18,19^. Hepatic ischemia reperfusion injury (HIRI) is a recognized complication of OLT and is strongly linked to EAD, PNF, longer hospital stays, and lower long-term graft survival ^18^. Therefore, strategies to attenuate HIRI may improve graft function and survival.

Dexmedetomidine, a selective α2-adrenoceptor agonist, is a useful adjuvant to general anesthesia which has sedative, anxiolytic, analgesic, and hypotensive properties. In vitro and in vivo preclinical studies have widely demonstrated that dexmedetomidine provides organ protection in kidney, lung, brain, heart, and liver tissues by ameliorating HIRI, inhibiting pro-inflammatory signaling pathways, and decreasing cell death ^20^ ^21^ ^22^ ^23^ ^24^ ^25^ ^26^. Regarding hepatic protection, both Chen et al. and Wang et al. have demonstrated that dexmedetomidine treatment protects against HIRI in rodents. Inhibition of the toll like receptor 4 (TLR4) nuclear factor kappa B (NF-κB) pathway has been implicated as one of the mechanisms underlying these protective effects ^27^. In the clinical setting, a randomized controlled trial by Wang et al. of 44 patients undergoing hepatectomy found that intraoperative treatment with dexmedetomidine (loading dose of 1 μg/kg over 10 min followed by a maintenance dose of 0.3 μg/kg/h) resulted in lower serum AST and ALT levels in the first 72 h postoperatively. However, the trial only looked at these biomarkers of hepatocellular damage and did not study patient and allograft outcomes ^28^. Likewise, a study by Fayed et al. of 40 patients undergoing living donor liver transplantation showed that intraoperative infusion of dexmedetomidine (0.8 μg/kg/h) improved postoperative liver function tests (on days 1, 3, and 5) and showed improved histopathological changes in liver tissue taken by biopsy at the end of surgery compared to controls. The authors did not look at long-term graft function or survival, and it is unclear whether these initial biochemical and histological benefits were associated with an improved clinical picture and better patient prognosis ^29^. Considering the strength of these preclinical data demonstrating the organ protective effects of dexmedetomidine, urgent randomized clinical trials are needed to evaluate its potentially beneficial impact in OLT. This study aimed to verify the hypothesis that the application of dexmedetomidine during the perioperative period of liver transplantation can reduce the incidence of EAD and PNF. At the same time, the effects of dexmedetomidine application on postoperative renal function and lung function were studied.

**Recruitment**

Inclusion criteria

Patients aged between 18 to 65 undergoing allogenic liver transplant surgery under general anesthesia, who meet the UCSF criteria (single tumor diameter ≤ 6.5 cm; multiple tumors ≤ 3, maximum diameter ≤ 4.5 cm; cumulative diameter ≤ 8 cm; without large vessel infiltration and extrahepatic metastasis) were eligible for trial inclusion.

Exclusion criteria

Patients with severe renal dysfunction (undergoing renal replacement therapy before surgery), severe pulmonary dysfunction (severe pre-existing chronic lung disease), severe circulatory instability (severe coronary artery disease, unstable angina, left ventricular ejection fraction < 30%, sick sinus syndrome, severe sinus bradycardia [< 50 bpm], second-degree or greater atrioventricular block), known allergy or intolerance to trial medication, participation in other clinical trials within 30 days prior to randomization, re-transplantation and multiple organ transplantation were excluded.

**Informed consent**

Informed consents were obtained from all participants. After receiving the operation notice, one designated anesthesiologist evaluated the patients, introduce this trial and answer questions. For every potential participant, the purpose, procedures, and potential benefits and risks of the study were explained in detail in a written informed manner. Participants’ right to withdraw consent at any time during the study period was also emphasized.

Potential participants were given at least a night to decide whether they wish participate. Every participant or the authorized surrogate of the participant signed the informed consent before they can be enrolled in the study. Written informed consents were kept as a part of the clinical trial documents.

**Randomization and blinding**

An independent statistician generated a random sequence of 0 and 1 in a 1:1 fashion correspondent to numbers from 1 to 330. Participants who consented to enter the trial were sequentially assigned a random number allocating them to either the dexmedetomidine or placebo group (normal saline). Caregivers were blinded to study arms. An independent pharmacist provided the trial drugs according to the allocation of patient group but did not participate in the rest of the study.

**Study procedures**

Anesthesia and intervention

All participants received standard anesthesia care according to our hospital’s protocol. Anesthesia was induced using propofol, sufentanil, and rocuronium or cisatracurium for neuromuscular blockage. Remifentanil, propofol and sevoflurane or desflurane were used for anesthesia maintenance. Mechanical ventilations were performed following routine protocol, in which tidal volume was kept between 6 and 8 ml/kg, positive end expiratory pressure (PEEP) at 5 cmH20, plateau pressure of less than 30 cmH20, ventilation frequency between 10 and 16 per minute, and 1:1 air-oxygen mixture. Intraoperative fluid therapy was managed according to the anesthesiologist’s routine practice. Hemoglobin levels were maintained within the range of 7–10 g/dL. Vasopressors were given when the anesthesiologist decided. Intravenous steroids were administered before reperfusion of the new liver as per normal protocol.

For the dexmedetomidine group, patients received an initial loading dose of dexmedetomidine of 1 μg/kg over 10min after the induction of anesthesia followed by a continuous infusion of 0.5 μg/kg/h until the end of surgery. In the control group, participants received a same volume of saline instead of dexmedetomidine in this group.

Allograft and surgery

Organ donation or transplantation in the study was strictly implemented under the regulation of Shanghai Organ Transplant Committee and the Declaration of Helsinki. Ethical approval was obtained from the Committee of Ethics at Ren Ji Hospital. Cadaveric donors involved in the study were brain-dead donors. Donors were preserved in conventional static cold preservation and transported to our medical center. All the surgical procedures were performed by specialists with experience in the LT technique at the Department of Liver Surgery, Ren Ji Hospital. Classic orthotopic liver transplantation (OLT) was the only surgical technique and postoperative care were given following our routine protocol.

Follow-up

Follow-up assessments were administered over phone at 3, 6, and 12 months. Cases of death, graft function and re-transplantation were recorded.

**Study outcomes**

Primary outcome

The primary outcome was the incidence of EAD following surgery, which is defined according to Olthoff’s criteria published in 2010: (1) bilirubin ≥10 mg/dL on day 7; or (2) INR > 1.6 on day 7; or (3) AST/ ALT > 2000 IU/L within first 7 days

Secondary outcomes

Secondary outcomes included incidence of primary nonfunction (PNF), incidence of postoperative acute kidney injury (AKI) and acute respiratory distress syndrome (ARDS) during postoperative day 1-7, incidence of graft failure and re-transplantation rate during follow-up period, all-cause mortality rate. PNF was defined as graft loss, re-transplantation, or participant’s death due to graft non-function in first 30 days (excluding non-function secondary to hepatic artery thrombosis, biliary complications, or recurrent hepatic disease). AKI was defined by Kidney Disease: Improving Global Outcomes (KDIGO) criteria published in 2012 and ARDS was defined according to Berlin modification of the American European Consensus Committee (AECC) definitions published in 2012.

**Data collection**

Data collection before surgery

Baseline data were collected after obtaining written informed consent. Including:

1). Demographic data: gender, date of birth, height, weight, and body mass index (BMI)

2). Medical data: diagnosis (reason for LT), comorbidities, concomitant medication, non-drug therapies, history of smoking and drinking, history of food or drug allergy, and history of anesthesia and surgery

3). Results of preoperative physical examinations

4). Baseline laboratory investigations

5). Donor characteristics: age, height, weight, BMI, cause of death, and virology status

Data collection during surgery

1). Duration of surgery, type of liver grafts (cadaveric or living), operating method

2). Duration of anesthesia and doses of anesthetic drugs given

3). Fluid balance during surgery, including estimated bleeding, type and volume of blood products transfused (document duration of storage before transfusion), and type and volume of fluid infusion

4). Variations of blood pressure and heart rate during anesthesia/surgery

5). Duration of cold and warm ischemia times

Data collection after surgery

Study outcomes were measured on day 1,3,7,14,30 and before discharge.

1). Incidence of EAD

2). Incidence of PNF

3). Incidence of AKI during postoperative days 1–7

4). Incidence of ARDS during postoperative days 1–7

5). Medication (including sedatives, analgesics, anticholinergics, and glucocorticoids) used during postoperative days 1–7 will be recorded

6). Length of stay in ICU and hospital after surgery

7). All-cause in-hospital and 30-day mortality

Long-term data collection

1). Incidence of graft failure and re-transplantation within 1 year

2). Status of survival. For participants who died after surgery, the date of death will be recorded.

**Addendum**

Based on our original study design, the plan was to follow participants for a period of 3 years after surgery ^30^ . Following the completion of the one-year follow-up, funding for this trial was halted. The Data and Safety Monitoring Board (DSMB) opted to analyze the data without disclosing the allocation. The subsequent statistical analysis revealed no significant difference between the two study arms. Specifically, the use of intraoperative dexmedetomidine, compared to normal saline, did not demonstrate a protective effect on early allograft dysfunction in liver transplant patients. These findings suggest that even with continued follow-up, it is highly unlikely that the intervention would show a significant benefit. As a result, the DSMB concluded to prematurely end the follow-up phase. Consequently, the DAS-OLT trial will only follow participants for up to 1 year.

1. D'Amico G, Garcia-Tsao G, Pagliaro L. Natural history and prognostic indicators of survival in cirrhosis: a systematic review of 118 studies. *J Hepatol.* 2006;44(1):217-231.

2. Disease GBD, Injury I, Prevalence C. Global, regional, and national incidence, prevalence, and years lived with disability for 310 diseases and injuries, 1990-2015: a systematic analysis for the Global Burden of Disease Study 2015. *Lancet.* 2016;388(10053):1545-1602.

3. Mortality GBD, Causes of Death C. Global, regional, and national life expectancy, all-cause mortality, and cause-specific mortality for 249 causes of death, 1980-2015: a systematic analysis for the Global Burden of Disease Study 2015. *Lancet.* 2016;388(10053):1459-1544.

4. Adam R, Karam V, Delvart V, et al. Evolution of indications and results of liver transplantation in Europe. A report from the European Liver Transplant Registry (ELTR). *J Hepatol.* 2012;57(3):675-688.

5. McCaughan GW, Munn SR. Liver transplantation in Australia and New Zealand. *Liver Transpl.* 2016;22(6):830-838.

6. Burra P, Samuel D, Sundaram V, et al. Limitations of current liver donor allocation systems and the impact of newer indications for liver transplantation. *J Hepatol.* 2021;75 Suppl 1:S178-S190.

7. Agopian VG, Harlander-Locke MP, Markovic D, et al. Evaluation of Early Allograft Function Using the Liver Graft Assessment Following Transplantation Risk Score Model. *JAMA Surg.* 2018;153(5):436-444.

8. Guo Z, Zhao Q, Jia Z, et al. A randomized-controlled trial of ischemia-free liver transplantation for end-stage liver disease. *J Hepatol.* 2023.

9. Golse N, Guglielmo N, El Metni A, et al. Arterial Lactate Concentration at the End of Liver Transplantation Is an Early Predictor of Primary Graft Dysfunction. *Ann Surg.* 2019;270(1):131-138.

10. Lee DD, Croome KP, Shalev JA, et al. Early allograft dysfunction after liver transplantation: an intermediate outcome measure for targeted improvements. *Ann Hepatol.* 2016;15(1):53-60.

11. Mazilescu LI, Kotha S, Ghanekar A, et al. Early Allograft Dysfunction After Liver Transplantation With Donation After Circulatory Death and Brain Death Grafts: Does the Donor Type Matter? *Transplant Direct.* 2021;7(8):e727.

12. Busuttil RW, Tanaka K. The utility of marginal donors in liver transplantation. *Liver Transpl.* 2003;9(7):651-663.

13. Hoyer DP, Paul A, Saner F, et al. Safely expanding the donor pool: brain dead donors with history of temporary cardiac arrest. *Liver Int.* 2015;35(6):1756-1763.

14. Deschenes M, Belle SH, Krom RA, Zetterman RK, Lake JR. Early allograft dysfunction after liver transplantation: a definition and predictors of outcome. National Institute of Diabetes and Digestive and Kidney Diseases Liver Transplantation Database. *Transplantation.* 1998;66(3):302-310.

15. Olthoff KM, Kulik L, Samstein B, et al. Validation of a current definition of early allograft dysfunction in liver transplant recipients and analysis of risk factors. *Liver Transpl.* 2010;16(8):943-949.

16. Croome KP, Marotta P, Wall WJ, et al. Should a lower quality organ go to the least sick patient? Model for end-stage liver disease score and donor risk index as predictors of early allograft dysfunction. *Transplant Proc.* 2012;44(5):1303-1306.

17. Chen XB, Xu MQ. Primary graft dysfunction after liver transplantation. *Hepatobiliary Pancreat Dis Int.* 2014;13(2):125-137.

18. Beck-Schimmer B, Breitenstein S, Urech S, et al. A randomized controlled trial on pharmacological preconditioning in liver surgery using a volatile anesthetic. *Ann Surg.* 2008;248(6):909-918.

19. Beck-Schimmer B, Breitenstein S, Bonvini JM, et al. Protection of pharmacological postconditioning in liver surgery: results of a prospective randomized controlled trial. *Ann Surg.* 2012;256(5):837-844; discission 844-835.

20. Kocoglu H, Karaaslan K, Gonca E, Bozdogan O, Gulcu N. Preconditionin effects of dexmedetomidine on myocardial ischemia/reperfusion injury in rats. *Curr Ther Res Clin Exp.* 2008;69(2):150-158.

21. Kocoglu H, Ozturk H, Ozturk H, Yilmaz F, Gulcu N. Effect of dexmedetomidine on ischemia-reperfusion injury in rat kidney: a histopathologic study. *Ren Fail.* 2009;31(1):70-74.

22. Gu J, Sun P, Zhao H, et al. Dexmedetomidine provides renoprotection against ischemia-reperfusion injury in mice. *Crit Care.* 2011;15(3):R153.

23. Wu Y, Liu Y, Huang H, et al. Dexmedetomidine inhibits inflammatory reaction in lung tissues of septic rats by suppressing TLR4/NF-kappaB pathway. *Mediators Inflamm.* 2013;2013:562154.

24. Alam A, Suen KC, Hana Z, Sanders RD, Maze M, Ma D. Neuroprotection and neurotoxicity in the developing brain: an update on the effects of dexmedetomidine and xenon. *Neurotoxicol Teratol.* 2017;60:102-116.

25. Yu X, Chi X, Wu S, et al. Dexmedetomidine Pretreatment Attenuates Kidney Injury and Oxidative Stress during Orthotopic Autologous Liver Transplantation in Rats. *Oxid Med Cell Longev.* 2016;2016:4675817.

26. Chi X, Wei X, Gao W, et al. Dexmedetomidine ameliorates acute lung injury following orthotopic autologous liver transplantation in rats probably by inhibiting Toll-like receptor 4-nuclear factor kappa B signaling. *J Transl Med.* 2015;13:190.

27. Chen Z, Ding T, Ma CG. Dexmedetomidine (DEX) protects against hepatic ischemia/reperfusion (I/R) injury by suppressing inflammation and oxidative stress in NLRC5 deficient mice. *Biochem Biophys Res Commun.* 2017;493(2):1143-1150.

28. Wang ZX, Huang CY, Hua YP, Huang WQ, Deng LH, Liu KX. Dexmedetomidine reduces intestinal and hepatic injury after hepatectomy with inflow occlusion under general anaesthesia: a randomized controlled trial. *Br J Anaesth.* 2014;112(6):1055-1064.

29. Fayed NA, Sayed EI, Saleh SM, Ehsan NA, Elfert AY. Effect of dexmedetomidine on hepatic ischemia-reperfusion injury in the setting of adult living donor liver transplantation. *Clin Transplant.* 2016;30(4):470-482.

30. Ni C, Masters J, Zhu L, et al. Study design of the DAS-OLT trial: a randomized controlled trial to evaluate the impact of dexmedetomidine on early allograft dysfunction following liver transplantation. *Trials.* 2020;21(1):582.

**Statistical Analysis Plan**

1. INTRODUCTION

DAS-OLT is a single-center, double-blinded, placebo-controlled randomized clinical trial intending to determine whether the use of dexmedetomidine reduces early allograft dysfunction after liver transplant. Eligible participants are allocated to dexmedetomidine airway or normal saline group in a 1:1 fashion.

The purpose of this statistical analysis plan is to outline the analyses and tests that will be used to answer the research objectives outlined in the DAS-OLT protocol and to explain how to analyses the data, and how to present the analysis results.

2. DATA SOURCE

The sources of data used for analysis are the electronic data capture (EDC) system, and the central randomization system. The EDC contains baseline and follow-up data while the randomization system contains allocation.

3. ENROLLMENT SUMMARY

The total number of enrolled patients, number of patients excluded from intention-to-treat (ITT) analysis or per-protocol (PP) analysis were presented.

4. BASELINE SUMMARIES

4.1 Definition and timing of baseline variables

Baseline variables are collected at the time of randomization, including demographic data (gender, date of birth, height, weight), medical data (diagnosis, reason for LT, comorbidities, concomitant medication, non-drug therapies, history of smoking and drinking, history of food or drug allergy, and history of anesthesia and surgery), results of physical examinations, baseline laboratory investigations, donor characteristics (age, height, weight, BMI, cause of death, and virology status).

Baseline laboratory assessments (blood routine, serum electrolyte level, arterial blood gas) are based on the most recent laboratory results before the surgery day.

4.2 Baselines summaries

Baseline demographic and clinical characteristics are tabulated by treatment group for the ITT population. For continuous variables, those normally distributed are presented as mean and standard deviation (SD); otherwise, they are presented as median and interquartile range. Categorical variables are demonstrated as count data with their percentages.

5. ANALYSIS OF PERIOPERATIVE CHARACTERISTICS

Intraoperative characteristics include anaesthesia time, surgery time, portal occlusion time, vena cava occlusion time, cold ischemia time, crystalloid and colloid solution infusion, blood loss during surgery, ascites amount administered. Statistical comparisons of the two randomized groups with respect to these variables are performed, using parametric or nonparametric test, whichever is appropriate.

6. ANALYSIS OF CLINICAL OUTCOMES

6.1 General

All statistical hypothesis tests will be two-sided and their precision (i.e., 95% confidence intervals) will be two-sided as well. Statistical comparisons of all clinical outcomes between the dexmedetomidine group and the normal saline group are based on intention-to-treat principle. Our intention-to-treat set excludes patients whose transplantation surgery was canceled, on which analyses are impossible to conduct.

6.2 Primary and secondary endpoints

The primary endpoint of this study is the EAD.

Key secondary outcomes are as follow:

PNF, incidence of AKI and ARDS during postoperative day 1-7, incidence of graft failure and re-transplantation rate during follow-up, all-cause mortality rate. PNF was defined as graft loss, re-transplantation, or participant’s death due to graft non-function in first 30 days (excluding non-function secondary to hepatic artery thrombosis, biliary complications, or recurrent hepatic disease). AKI was defined by Kidney Disease: Improving Global Outcomes (KDIGO) criteria published in 2012 and ARDS was defined according to Berlin modification of the American European Consensus Committee (AECC) definitions published in 2012.

6.3 Analysis of the endpoints

The effect of dexmedetomidine and normal saline on EAD was compared using the Chi-squared test, and its relative risk was estimated by Wald maximum likelihood estimation with corresponding 95% CIs.

Like the primary endpoint, for other binary secondary outcomes, we will conduct comparisons using maximum likelihood estimation and Chi-squared tests. Continuous outcomes will be analyzed using the t-test or Kruskal-Wallis rank-sum test according to their distribution, which will be determined by the Kolmogorov-Smirnov test.

6.4 Subgroup analysis and assessment of covariate-by-treatment interactions

Subgroup analysis along with a covariate-by-treatment interaction test will be performed with respect to the following strata:

Age (>50 or ≤50);

Sex;

Child-Pugh score;

MELD score;

Portal occlusion time;

Cold ischemia time;

Liver condition;

Blood transfusion.
